# The paradoxes hidden behind the Droop model highlighted by a metabolic approach

**DOI:** 10.3389/fpls.2022.941230

**Published:** 2022-08-22

**Authors:** Caroline Baroukh, Francis Mairet, Olivier Bernard

**Affiliations:** ^1^LIPME, Université de Toulouse, INRAE, CNRS, Castanet-Tolosan, France; ^2^Ifremer, PHYTOX, Laboratoire PHYSALG, Nantes, France; ^3^Biocore, INRIA, Université Côte d'Azur, Sophia Antipolis, France

**Keywords:** metabolic network, microalgae, nitrogen stress, excretion, *Tisochrysis lutea*

## Abstract

We propose metabolic models for the haptophyte microalgae *Tisochrysis lutea* with different possible organic carbon excretion mechanisms. These models—based on the DRUM (Dynamic Reduction of Unbalanced Metabolism) methodology—are calibrated with an experiment of nitrogen starvation under day/night cycles, and then validated with nitrogen-limited chemostat culture under continuous light. We show that models including exopolysaccharide excretion offer a better prediction capability. It also gives an alternative mechanistic interpretation to the Droop model for nitrogen limitation, which can be understood as an accumulation of carbon storage during nitrogen stress, rather than the common belief of a nitrogen pool driving growth. Excretion of organic carbon limits its accumulation, which leads to a maximal C/N ratio (corresponding to the minimum Droop N/C quota). Although others phenomena—including metabolic regulations and dissipation of energy—are possibly at stake, excretion appears as a key component in our metabolic model, that we propose to include in the Droop model.

## 1. Introduction

Microalgae are unicellular eukaryotic microorganisms, playing a key role in the ocean. How nutrient stress affects microalgal growth is a central issue, particularly in the context of climate change (Moore et al., 2013). Microalgae are also promising sources of products, addressing various markets including animal feeding (aquaculture, poultry or pig farming), green chemistry (food colorants) or biofuel (Spolaore et al., 2006; Mata et al., 2010; Wijffels and Barbosa, 2010). Nutrient stress can be used to trigger product accumulation (e.g., to increase neutral lipid content for biofuel production), but growth is severely hindered during these adverse growing conditions (Lacour et al., 2012a; Huang et al., 2019). Better understanding the response of microalgal metabolism to dynamical conditions (of light, nutrients, etc.) is therefore key to understand their dynamics in their natural environment but also to tame them for biotechnological applications.

Systems biology and metabolic modeling have proven to be very efficient tools for helping to understand microorganisms' metabolism. Indeed, *in silico* studies can help to clarify the intracellular mechanisms taking place and open routes for optimizing the production of molecules of interest (Kim et al., 2017). Systems biology can pave the way toward a better comprehension of microalgae metabolism during nitrogen starvation. So far, metabolic modeling of photosynthetic microorganisms have mainly focused on balanced growth (Tibocha-Bonilla et al., 2018), although some recent promising developments deals with dynamical conditions (e.g., Broddrick et al., 2016; Flassig et al., 2016; Loira et al., 2017; Reimers et al., 2017; Zuniga et al., 2018; Sarkar et al., 2019). However, no metabolic model exists to represent and understand nitrogen starvation under day/night cycles in microalgae. Only macroscopic models were used so far, see e.g., Geider et al. (1998) and Muñoz-Tamayo et al. (2013). Most of them are based on the Droop model (Droop, 1968, 1983). This empiric model is widely used to predict microalgal behavior under nutrient limited conditions. It represents the specific growth rate μ as a function of the intracellular quota of the limiting nutrient *q*


(1) $$\begin{array}{r} \mu \left(q\right)=\mu_{m}\left(1-\frac{Q_{0}}{q}\right) \end{array}$$

where $\bar{\mu }$ is the growth rate at an hypothetical infinite quota, while *Q*_0_ is the minimum quota for phytoplankton growth. Yet, the Droop model and all its derivatives rely on empiric laws that do not allow representing the intracellular mechanisms at place.

In this article, we focus on *Tisochrysis lutea*—a haptophyte widely used in aquaculture and considered for fucoxanthin or biofuel productions (Bendif et al., 2013; Garnier et al., 2014; Mohamadnia et al., 2020)—whose core metabolic network (Baroukh et al., 2014) allows to represent the synthesis of its main constituants (proteins, lipids, carbohydrates, RNA, DNA and chlorophyll). A metabolic model is used to study the dynamics of carbon storage accumulation during nitrogen stress. More precisely, five competitive models were built, from a metabolic model developed for non-limiting nitrogen conditions (Baroukh et al., 2014), by adding extensions implementing various excretion pathways which are generally neglected. These models were calibrated and validated by comparing their predictions to experimental data of respectively a nitrogen starvation under day/night cycles (Lacour et al., 2012a) and nitrogen limited chemostat equilibria (Lacour et al., 2012b). Once validated, these models provide a paradoxical interpretation of the Droop model.

## 2. Results

### 2.1. A metabolic model without excretion

**Figure 1:**
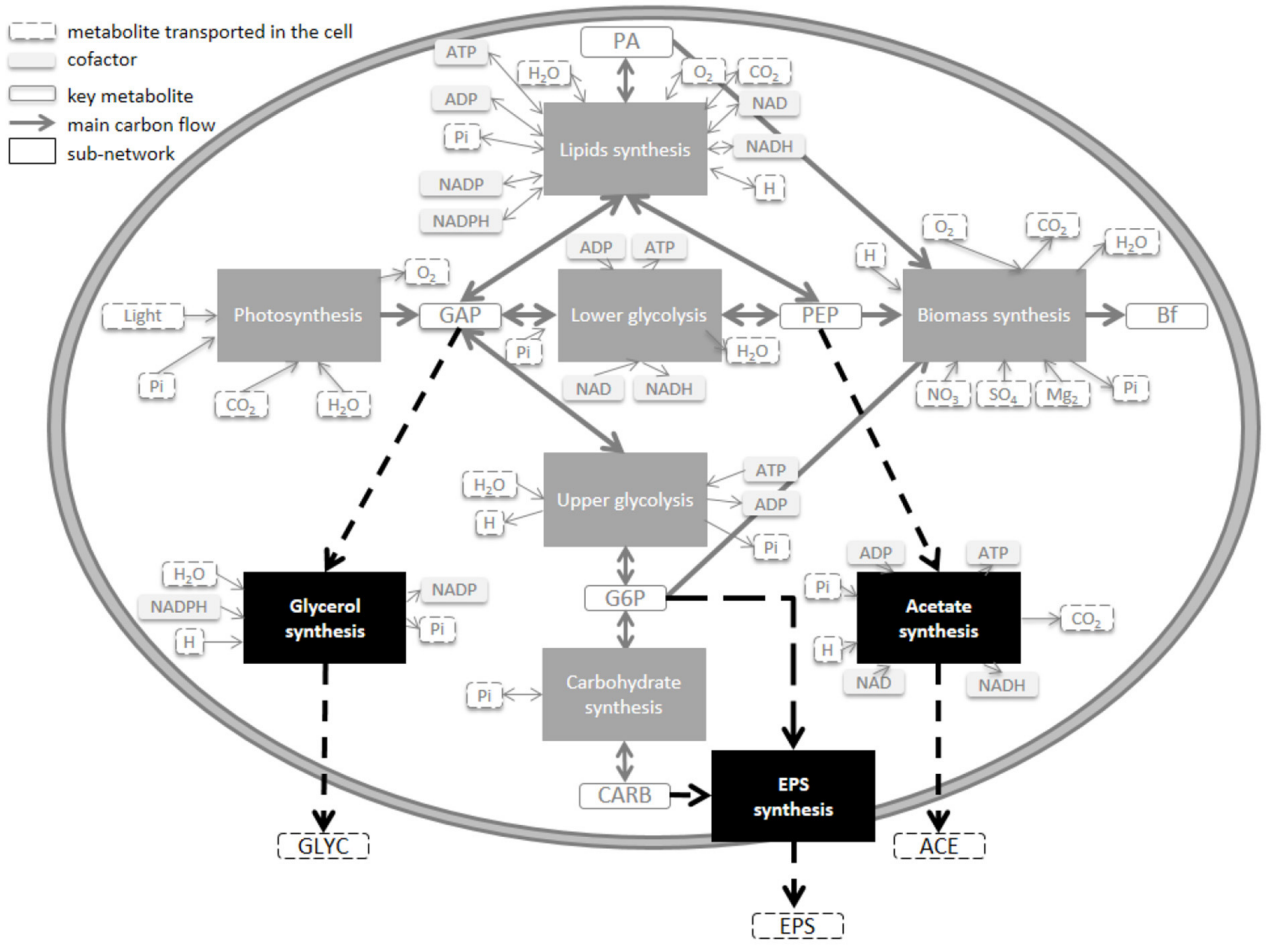
Model of the central carbon metabolic network of a unicellular photoautrotophic microalgae including different carbon excretion extensions. Three excreted molecules (EPS, ACE, and GLYC) were tested at four different loci of the metabolism (CARB, G6P, PEP, and GAP).

Microalgae submitted to day/night cycles exhibit an unbalanced growth: they accumulate lipids and carbohydrates during the day to support cell division and maintenance during the night (Lacour et al., 2012a; Vitova et al., 2015). The DRUM (Dynamic Reduction of Unbalanced Metabolism) framework was used to deal with unbalanced growth. The idea is to split the metabolic network into subnetworks, in which no internal compounds can accumulate (Baroukh et al., 2014). But, unlike the classical quasi-steady-state frameworks, some metabolites can accumulate when situated at the junction between the subnetworks. These subnetworks are defined by metabolic functions, taking into account cell compartments and metabolic pathways. Here we use a core metabolic network of phototrophic eukaryote, developed for *T. lutea* (Baroukh et al., 2014). This network, composed of 157 metabolites and 162 reactions, as most of the concurrent metabolic models of microalgae (Baroukh et al., 2015), neglect the loss of carbon due to excretion [except in the theoretical study of Ofaim et al. (2021)]. We split the network into six subnetworks corresponding to Figure 1: i) photosynthesis ii) upper glycolysis iii) lower glycolysis iv) carbohydrates synthesis v) lipids synthesis vi) functional biomass synthesis (composed of DNA, RNA, proteins, chlorophyll and membrane lipids). Metabolites that can internally accumulate (A) are glyceraldehyde 3-phosphate (GAP), glucose 6-phosphate (G6P), phosphoenolpyruvate (PEP), neutral lipids (represented by phosphatidic acid, PA) and carbohydrates (CARB). Functional biomass (B) is defined as the total biomass (Xc) without the compounds which do accumulate. Each sub-network is then reduced to macroscopic reactions (MRs) thanks to elementary flux mode analysis (Table 1). When a subnetwork has more than two elementary flux modes, the one with the best carbon yield was selected. Seven MRs were obtained (represented as a stoichiometric matrix K'). A mass action law hypothesis was used for the kinetics (α), assumed proportional to the product of the intracellular concentration of the metabolites necessary for the reaction (i.e., proportional to $\prod \frac{A_{i}}{B}$), or to reactor concentration for external substrates (Table 1). While each sub-network has a fixed stoichiometry, the different kinetic rates lead to specific dynamics for each metabolites A, and therefore ultimately to a variable biomass composition. The resulting metabolic model is called w/oEx (standing for “without excretion”), see Section 4 for more details.

**Table 1:** Sub-networks and resulting macroscopic reactions and kinetic rates.

	**Sub-network**	**Macroscopic reactions**	**Kinetic rates**
MR1	Photosynthesis	30 Light +3 CO_2_ +2 H_2_O +Pi → GAP +3 O_2_	*k*_MR1_*I*(*t*)
MR2	Upper glycolysis	2 GAP +H_2_O → G6P +Pi	*k*_MR2_ GAP/B
MR2'		G6P +ATP → H +ADP +2 GAP	$k_{\text{MR2}}^{\prime }$ G6P/B
MR3	Lower glycolysis	GAP +ADP +Pi +NAD ↔ PEP +ATP	*k*_MR3_ GAP/B - $k_{\text{MR3}}^{\prime }$ PEP/B
		+NADH +H_2_O +H	
MR4	Carbohydrate synthesis	G6P ↔ CARB +Pi	*k*_MR4_ G6P/B - $k_{\text{MR4}}^{\prime }$ CARB/B
MR5	Lipids synthesis	GAP +16.61 PEP +2 ADP +13.46 NAD +29.3 NADPH	*k*_MR5_ GAP/B . PEP/B - $k_{\text{MR5}}^{\prime }$ PA/B
		+34.48 H +2.15 O_2_↔ PA +14.61 Pi +2 ATP	
		+13.46 NADH +29.3 NADP +4.31 H_2_O +16.61 CO_2_	
MR6	Biomass synthesis	3.13 PEP +7.37 O_2_ +4.46 H +1.31 NO_3_ +1.14 G6P	*k*_MR6_ PEP/B . G6P/B . PA/B . NO3
		+0.11 PA +0.03 SO_4_ +0.0025 Mg → B	
		+11.67 CO_2_ +4.23 Pi +6 H_2_O	
MR7	Excretion	i) CARB → EPS	*k*_EX_ CARB/B
		ii) G6P → EPS +Pi	*k*_EX_ G6P/B
		iii) PEP +NAD +2 ADP +Pi +H → ACE +CO_2_	*k*_EX_ PEP/B
		+NADH +2 ATP	
		iv) GAP +H_2_O +H +NADPH → GLYC +Pi +NADP	*k*_EX_ GAP/B

### 2.2. The model without excretion overestimates carbon fixation under nitrogen starvation

**Figure 2:**
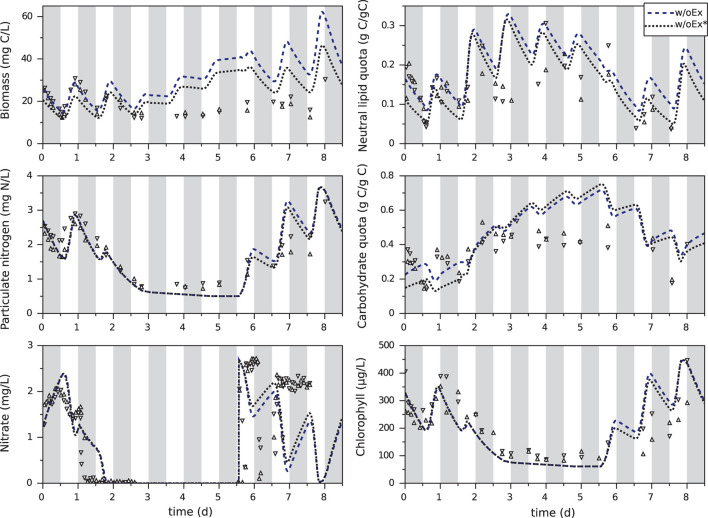
Calibration of the original model with the first day (w/oEx) or the whole experiment (w/oEx*) of *T. lutea* culture under day/night cycles and nitrogen starvation. Experimental data taken from Lacour et al. (2012a) are represented by symbols. Gray bands represent night periods.

Experimental data of *T. lutea* culture under day/night cycles (Lacour et al., 2012a) were used to estimate the 10 kinetic parameters (see Section 4). Fitted only on the first day, the w/oEx model is not able to correctly predict the experimental data when nitrogen is exhausted (Figure 2). Total organic carbon biomass *X*_*C*_ is overestimated from day 3 up to twofold at day 6, just before the end of nitrogen starvation. On the other hand, total organic nitrogen biomass *X*_*N*_ and chlorophyll are globally well predicted. The parameters of the w/oEx model were estimated using data from nitrogen replete period only, which might explain the discrepancy of the model to represent the experimental data during nitrogen starvation. Therefore, a new parameter estimation (w/oEx*) was performed on the whole set of data, including both nitrogen replete and nitrogen deplete conditions. A better fit was found, with lower error (-27,2%) and lower standard deviations on parameters (Table 2). Nevertheless carbon biomass is underestimated at the beginning of the experiment, and then overestimated during nitrogen starvation (slightly less than with the original parameter values). The new set of parameters achieves a trade-off by underestimating carbon fixation during nitrogen replete conditions not to dramatically overestimate carbon accumulation during nitrogen deplete conditions. Even with the new set of parameters, the w/oEx* model does not accurately represent the data for both nitrogen replete and stress conditions. The overestimation of TAGs, carbohydrates and total organic carbon by the model obtained from non-limiting conditions (Figure 2) is probably due to the unmodeled excretion of some carbon compound such as exopolysaccharides (EPS), glycerol or acetate during nitrogen starvation. In the next section, this hypothesis was tested *in silico* to identify its ability and relevance for describing the microalgae metabolism under nitrogen starvation.

**Table 2:** Estimated kinetic parameters (with their standard deviations) and resulting least squared error.

**Parameter (unit)**	**w/oEx**	w/oEx*	**Ex CARB**	**Ex G6P**	**Ex PEP**	**Ex GAP**
*k*_MR1_ (10^−3^ μE^−1^.m^2^.s.h^−1^. mol B^−1^)	1.30 ± 0.02	1.10 ± 0.01	1.61 ± 0.02	2.11 ± 0.04	4.50 ± 0.18	4.14 ± 0.19
*k*_MR2_ (mol^−1^.h^−1^)	205 ± 1,761	62.6 ± 112.9	6.07 ± 4.55	3.32 ± 1.43	569 ± 195	0.589 ± 0.069
$k_{\text{MR2}}^{\prime }$ (mol^−1^.h^−1^)	0.216 ± 73.3	0 ± 0.89	0.092 ± 1.41	0 ± 0.47	3.04 ± 2.44	2.62 ± 2.0
*k*_MR3_ (10^3^ mol^−1^.h^−1^)	8.10 ± 67.6	7.70 ± 20.1	7.96 ± 7.21	0.347 ± 0.141	5.17 ± 0.94	0.0262 ± 0.011
$k_{\text{MR3}}^{\prime }$ (mol^−1^.h^−1^)	11.9 ± 237	321 ± 1,548	547 ± 964	122 ± 120	0 ± 3.2	36.2 ± 11.4
*k*_MR4_ (mol^−1^.h^−1^)	16.8 ± 110	13.5 ± 15.7	13.6 ± 7.4	46.2 ± 19.4	13,400 ± 8,140	403 ± 254
$k_{\text{MR4}}^{\prime }$ (mol^−1^.h^−1^)	0 ± 0.11	0 ± 0.001	0 ± 0.01	1.21 ± 0.77	376 ± 248	16.4 ± 10.0
*k*_MR5_ (mol B.mol^−2^.h^−1^)	55.9 ± 471	141 ± 280	0.783 ± 0.470	0.413 ± 0.12	108 ± 37	0.0967 ± 0.0340
$k_{\text{MR5}}^{\prime }$ (10^−3^ mol^−1^.h^−1^)	39.3 ± 28.3	43.1 ± 8.9	120 ± 15	117 ± 9.9	90.0 ± 9.6	106 ± 8.1
*k*_MR6_ (10^3^mol B^2^.mol^−4^.h^−1^)	11.5 ± 99.4	317 ± 195	2.89 ± 1.38	1.34 ± 0.97	2.03 ± 1.52	1.17 ± 0.93
*k*_EX_ (mol^−1^.h^−1^)	-	-	0.0778 ± 0.0040	5.90 ± 4.68	7.29 ± 1.24	3.47 ± 0.27
**Calibration**						
Least squared error	58.5	46.0	33.4	34.2	33.0	34.4
% improvement	–27.2	0.	27.4	25.7	28.3	25.2
AICc	–439	–508	–598	–592	–602	–590
ATP unbalance (%)	–4.37	–4.16	–3.23	–2.57	–19.6	–0.67
NADPH unbalance (%)	6.98	6.51	3.95	3.22	1.61	13.7
**Validation**						
Least squared error		1.44	1.15	1.13	1.64	1.28
% improvement		0.	20.1	21.7	–13.8	11.4

### 2.3. Including excretion in the metabolic model

As for all the existing microalgal metabolic models, no excretion pathway was included in the original metabolism. However, excretion was observed for several microalgae species (Claquin et al., 2008; Szul et al., 2019), particularly during nutrient deplete conditions (Staats et al., 2000; Underwood et al., 2004; Szul et al., 2019). The objective was thus to *in silico* assess how various excretion scenarii are likely to modify the metabolic fluxes and eventually improve the predictive capacities of the model.

Excretion is species-dependent (Hellebust, 1965), and the nature of the excreted molecules for *T. lutea* is unknown. Three different common organic molecules were tested *in silico*, at four different levels of the metabolic network (Figure 1): i) exopolysaccharides-like molecules from carbohydrates (CARB) or glucose 6-phosphate (G6P), ii) acetate (ACE) and iii) glycerol (GLYC). Each tested metabolite was linked to an accumulating metabolite (PEP, GAP, G6P, and CARB) of the w/oEx model, since each metabolite is only few reactions steps (maximum 3) away. Each excretion pathway was assumed to be a new sub-network from which four macroscopic reactions were deduced (Table 1, see also Section 4.2 for details). Mass action kinetics were assumed, in line with the kinetics used for the w/oEx model. Only a single excretion pathway was tested at a time and four models were obtained: i) CARB excretion (ExCARB) ii) G6P excretion (ExG6P) iii) PEP excretion (ExPEP) and iv) GAP excretion (ExGAP).

### 2.4. Models with excretion do capture microalgal dynamics

The four modified models include excretion of a metabolite (EPS, ACE, or GLYC), at different loci of the metabolism (Figure 1). Parameters were re-identified for each model so as to find the best set of parameters that could fit the experimental data (Table 2).

**Figure 3:**
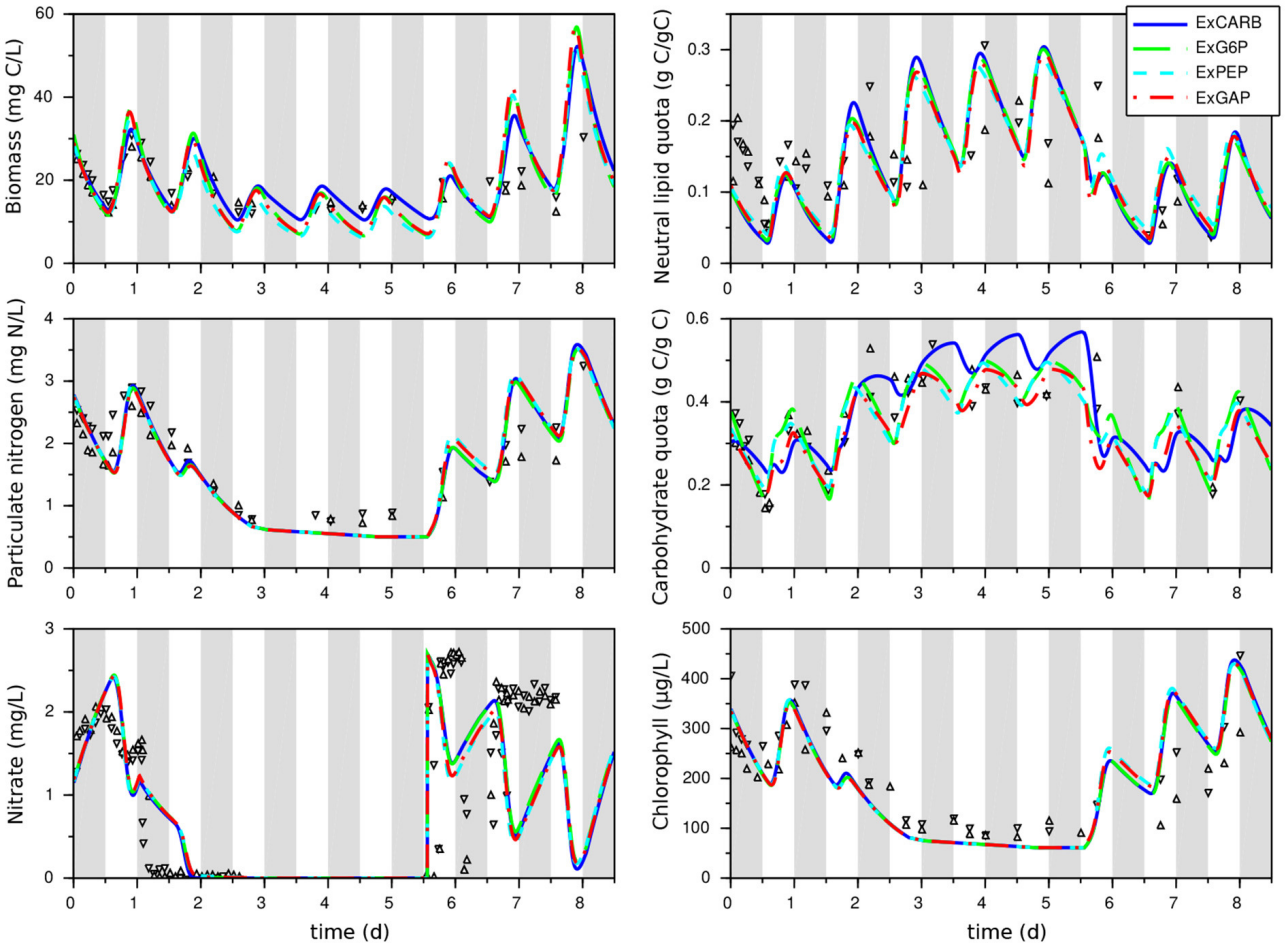
Calibration of the models that take into account excretion with a *T. lutea* culture under day/night cycles and nitrogen starvation. Experimental data taken from Lacour et al. (2012a) are represented by symbols. Gray bands represent night periods.

The models for each excretion hypothesis correctly fit the experimental data for both nitrogen replete and nitrogen starvation conditions (Figure 3 and Table 2), showing a real improvement in comparison with the model without excretion (as reflected by lower AICc). The confidence intervals on model parameters are also globally reduced, as well as the uncertainties on model outputs (see Supplementary Figure S2). Day/night accumulation and reuse of lipids and carbohydrates are well represented, even if neutral lipids are underestimated in nitrogen replete conditions. The simulated dynamics are almost the same for the four models, except for the carbohydrate quota which are higher for the ExCARB model during nitrogen starvation. It is therefore difficult to discern, between the four excretions, the most appropriate one. All the models present similar fittings, the lowest least square error being obtained with ExPEP in this calibration step.

**Figure 4:**
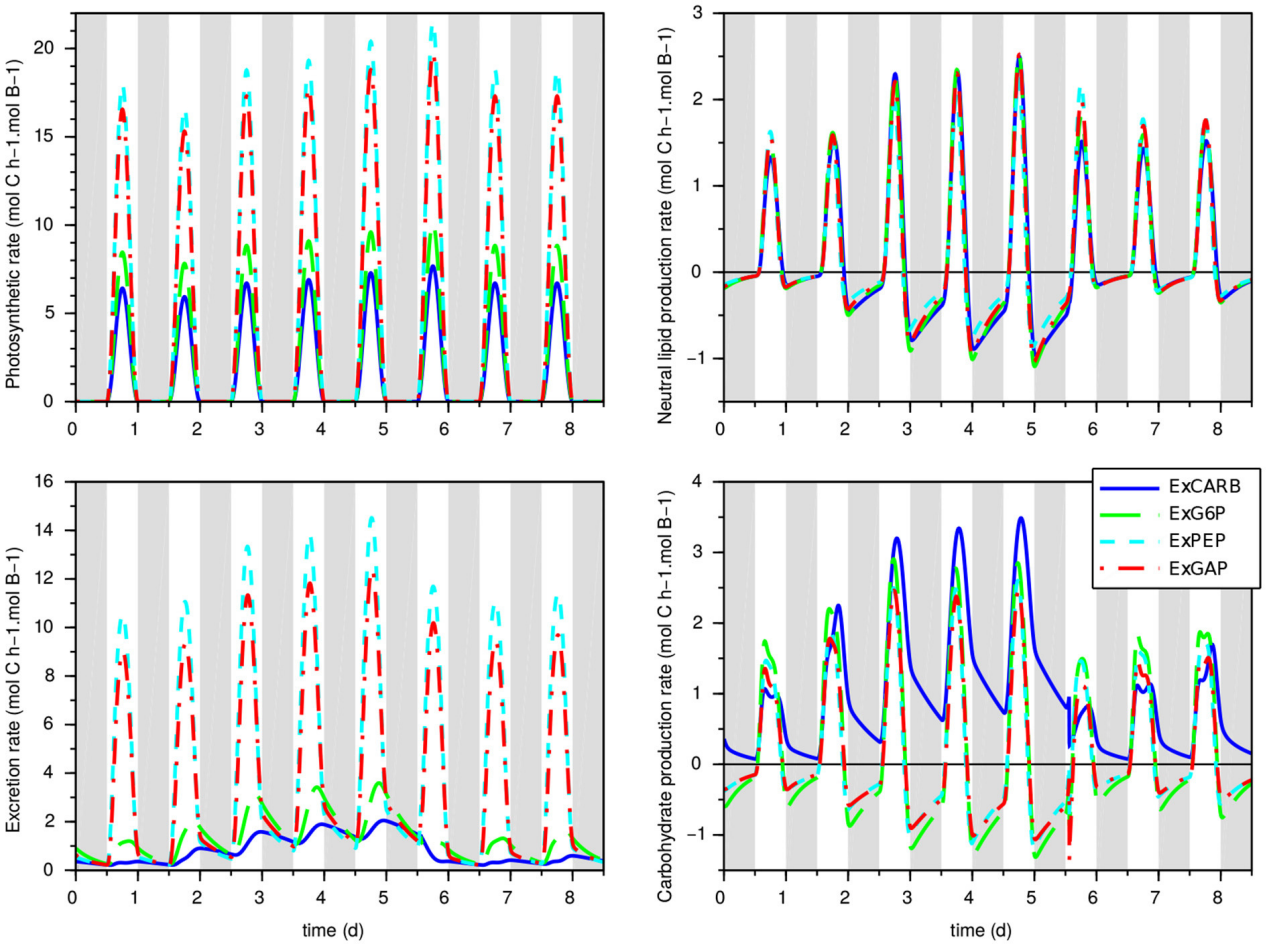
Prediction of carbon flux rates for the models with excretion in the calibration experiment. Top left: photosynthetic rate; bottom left: excretion rate; top right: neutral lipid production; bottom right: carbohydrate production. Gray bands represent night periods.

The clear fit improvement when considering excretions (compared to the w/oEx* model) gives some insights of the impact of nitrogen starvation on the metabolism of *T. lutea*. The four models predict different metabolic modes depending on light and nitrogen availability. What differs is the glycolysis direction, the carbon storage direction (consumption or accumulation of lipids and carbohydrates) and the relative distribution between carbon storage sources, photosynthesis and excretion. First, as expected, the excretion rates dictated by the intracellular metabolite concentrations increase during nitrogen starvation (see Figure 4). Although the four models show quite similar behavior in term of biomass prediction, they give different estimation of the amount of excreted carbon, compensated by different fluxes of carbon fixation. ExGAP and ExPEP predict the highest excretion rates (associated with the highest photosynthetic rates).

The intracellular fluxes also change, depending on the locus of excretion. Metabolic fluxes show that, during the day, the four models present different allocations of the assimilated inorganic carbon between functional biomass, carbon storage and excretion. During the night, excretion is almost null in nitrogen replete condition, while it continues—at a lower level—during nitrogen starvation (Figure 4). Night excretion is fuelled by carbon storage (mainly neutral lipids). Note that when the locus of excretion is CARB, the synthesis rate of carbohydrate is positive during the night due to the carbon flux from lipids to EPS, resulting in an upper glycolysis in the glyconeogenic direction. Although fatty acids β-oxidation coupled with gluconeogenesis is common (Kong et al., 2018), its occurrence at night needs to be confirmed experimentally.

**Figure 5:**
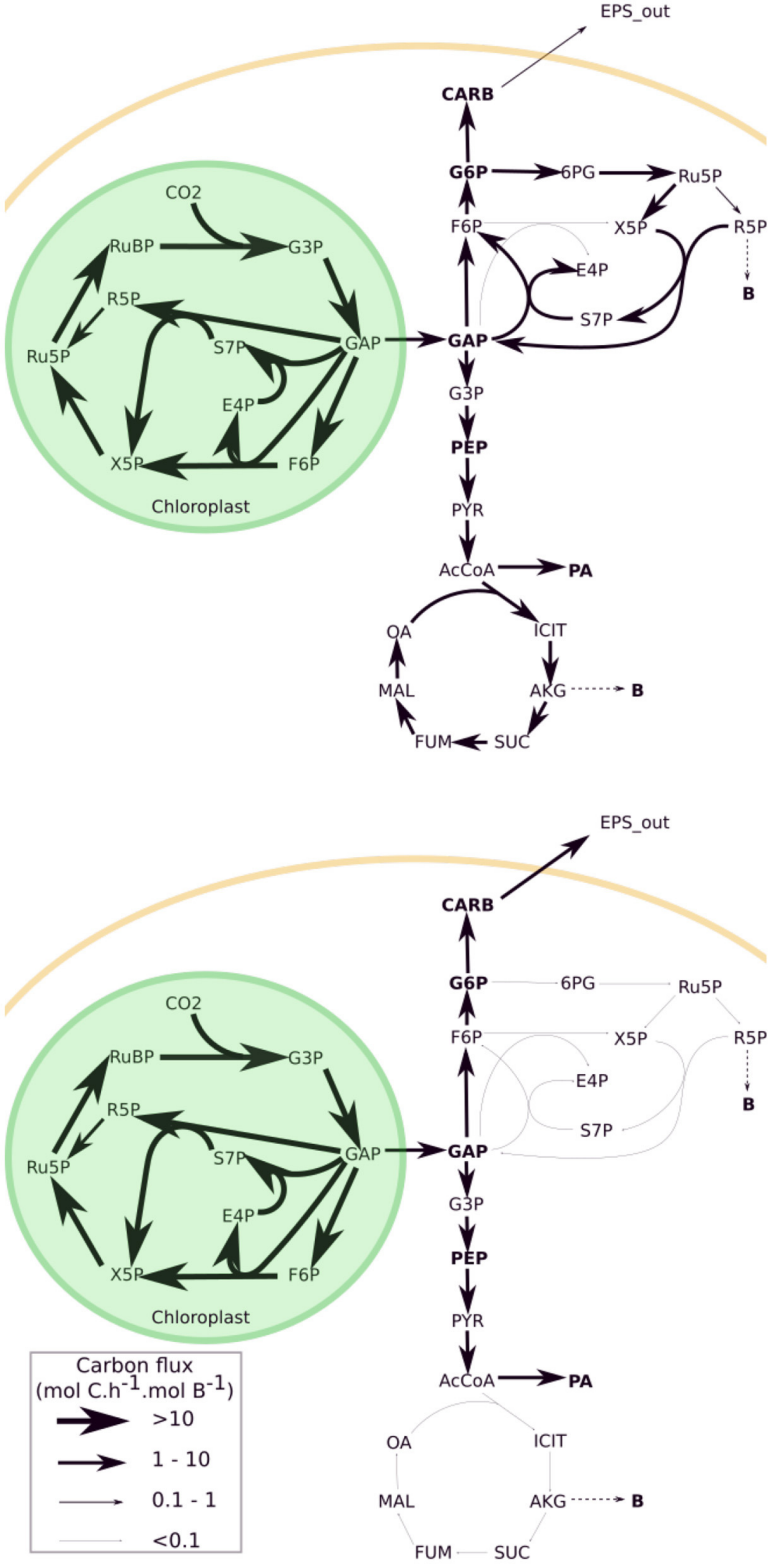
Metabolic fluxes obtained with the ExCARB model at noon for nitrogen replete (top, *t* = 0.75 d) and starved (bottom, *t* = 3.75 d) conditions. The conversion between flux map is given is the legend box of the figure (the same conversion was used for both maps). Bold metabolites indicate metabolites A allowed to accumulate. The complete names of metabolites can be found in the list of abbreviations.

During nitrogen starvation, the flux maps differ greatly from the nitrogen replete conditions. Except the Calvin cycle whose fluxes (per unit of functional biomass) remain almost unchanged, all the other fluxes are much lower and, as expected, some parts of the metabolic network are not activated (see Figure 5 and Supplementary File S2). As there is no functional biomass synthesis, the pentose phosphate pathway and the TCA cycle are not active. This result might be artificial because lipid synthesis requires NADPH reductive power and hence potentially requires the pentose phosphate pathway. Still, NADPH can be directly synthesized from photophosphorylation, if lipids synthesis takes place in the chloroplast as it has been hypothesized (Boyle and Morgan, 2009).

**Figure 6:**
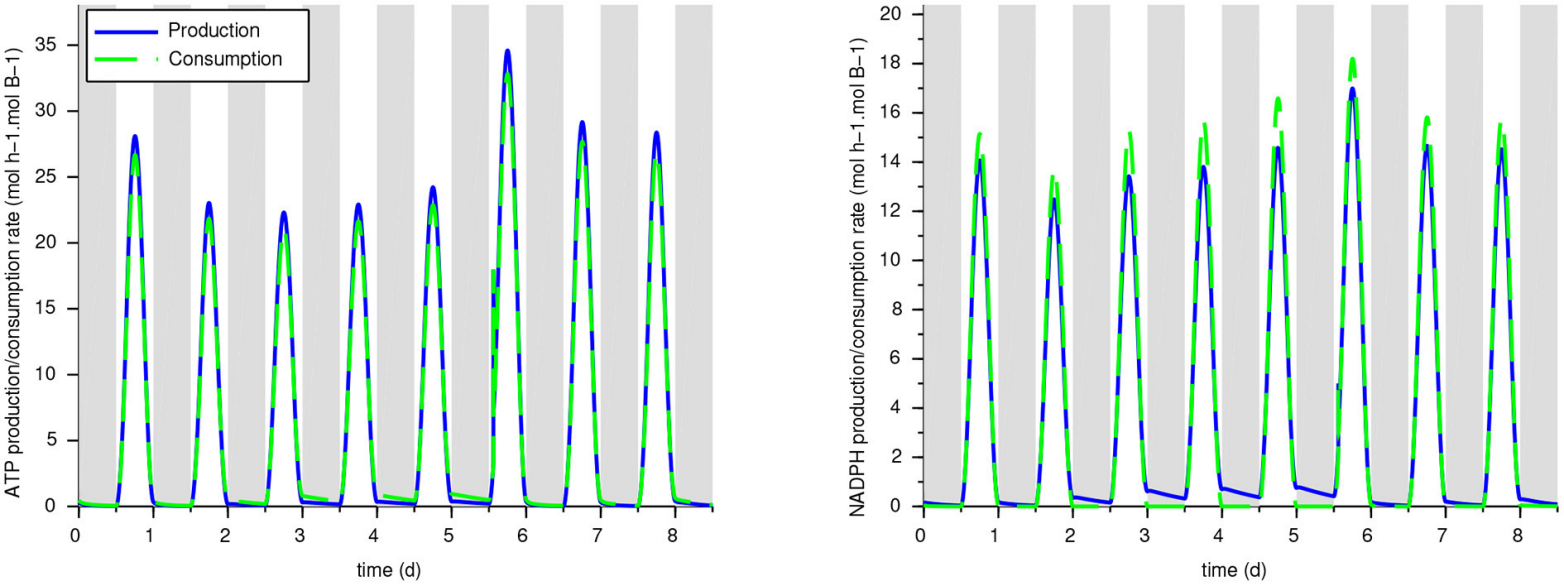
Production and consumption rates for ATP and NADPH with the ExCARB model. The ATP and NADPH balances—not imposed in the model—are validated a posteriori.

Our reduced model was developed with a focus on C and N fluxes, without constraining ATP and NADPH balance which should be a model outcome. Some subnetworks such as MR6 are perfectly balanced (so ATP and NADPH do not appear in the macroscopic reaction), but others are not, meaning that should exchange cofactors with other subnetworks. To validate the energy balance in the model, we computed the production and consumption rates of ATP and NADPH from the reactions of the whole metabolic network. It results that for w/oEx*,Ex CARB and Ex G6P, the ATP and NADPH are globally well balanced (see Figure 6 and Table 2): the relative difference between the overall consumption and the production do not exceed 7%. This means that the underlying hypotheses lead to a realistic energy balance in the cell. On the contrary, ATP is produced in excess in the ExPEP model and, NADPH is consumed in excess in the ExGAP model, pointing out that these hypothetical metabolic modes would require additional mechanisms to be included in our current models (such as cyclic electron flow) to balance their cofactors.

Lipid and carbohydrate quotas reach an almost periodic regime at day 3 (Figure 3). These values are higher than the maximal value reached during a day/night cycle in nutrient replete conditions, but they do not increase anymore after day 3. Hence, in these conditions, a long nitrogen starvation is not a good strategy for improving lipid and carbohydrate production yields, given that most of the carbon fixed during the day is excreted during the night.

### 2.5. Model validation in nitrogen-limited balanced growth conditions

**Figure 7:**
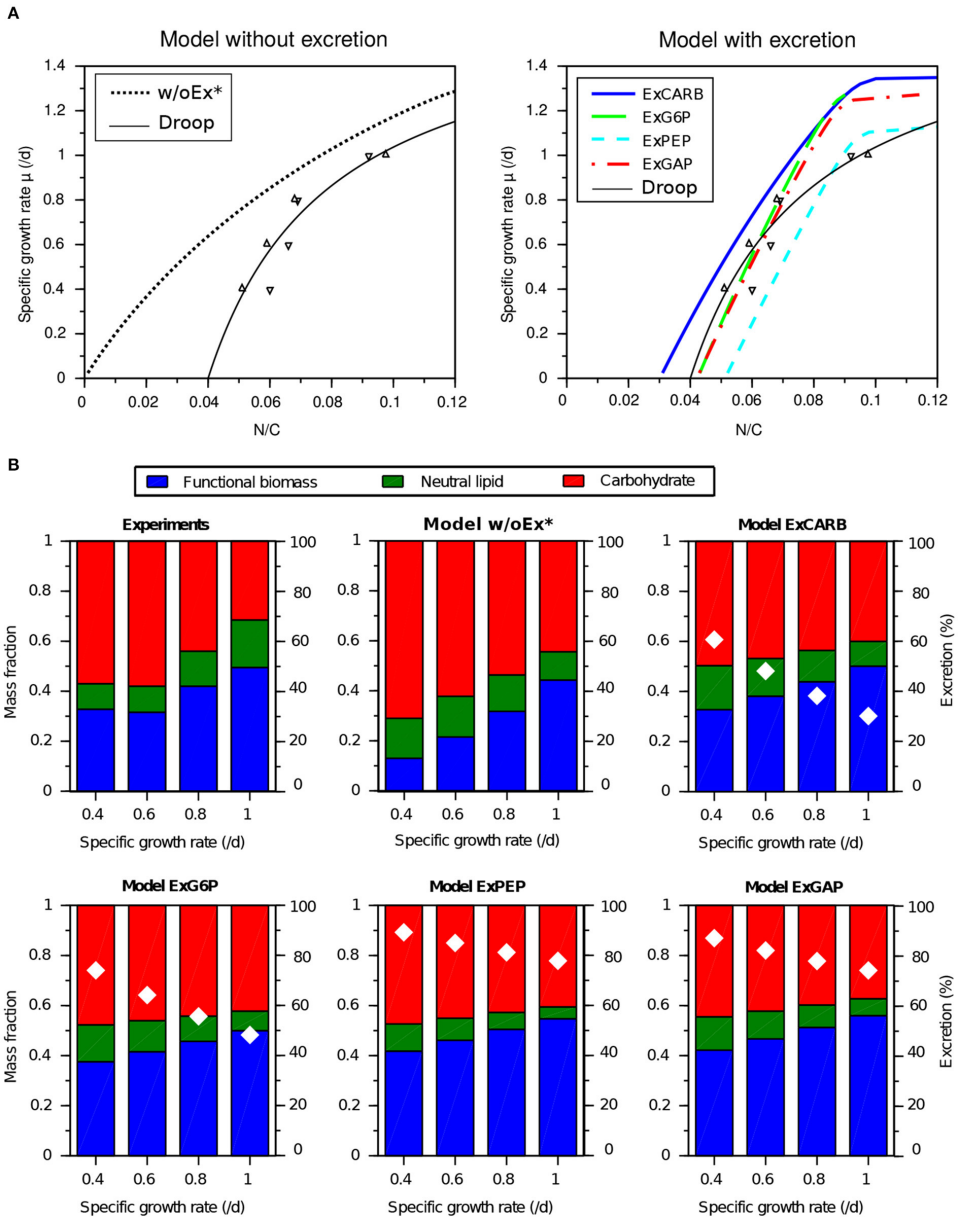
Experimental observations and predictions of *T. lutea* growth and biochemical composition under nitrogen-limited balanced growth conditions (with constant light). Experimental data of different nitrogen-limited equilibria in chemostat (Lacour et al., 2012b) are used for model validation. **(A)** Specific growth rate as a function of the nitrogen quota. **(B)** Biochemical composition (color bars) and percentage of excreted carbon over total carbon fixed (white diamonds) as a function of the specific growth rate.

After the calibration with starvation cultures under day/night cycles, the parameter were kept constant to assess the predictive capabilities of the four models with steady-states nitrogen-limited conditions under constant light. All the models predict an increasing relationship between the nitrogen quota (N:C) to the specific growth rate (Figure 7A). For the model without excretion w/oEx*, the curve starts at the origin. This behavior does not comply with the minimum quota for growth usually observed (Droop, 1968). All the models with excretion predict a minimum quota and are consistent with the experimental data at steady state, except for the highest quota for which the growth rate is overestimated. This discrepancy may be explained by the fact that the calibration was performed under day/night cycles, while this new experiment was carried out under continuous light. It is worth noting that the models simulate growth rates close to the one predicted by a standard Droop model (Equation 1) fitted with these data (leading to $\bar{\mu }$ = 1.73 d^−1^ and *Q*_0_ = 0.04 gN.gC^−1^), although the saturation is less pronounced with the latter. In addition, the models also predict an almost linear decreasing relationship between the specific growth rate and carbon storage (Figure 7B). For all the models with excretion, this prediction is also consistent with the experimental data-set, although neutral lipids are slightly underestimated at high growth rates. The percentage of fixed carbon which is excreted also decreases with growth rate, ranging e.g., for the ExCARB model from 60 to 25% when growth rate increases from 0.4 to 1 d^−1^.

Considering at the same time calibration and validation steps, the models considering EPS excretion (ExCARB and ExG6P) give the best results. The ExPEP model presents a lower performance in validation, while the NADPH was not balanced with the ExGAP model in the day-night simulation. Additionally, the ExPEP and ExGAP models must be discarded due to their unlikely high rate of excretion (see Figure 4).

## 3. Discussion

### 3.1. Carbon excretion as a crucial component of *T. lutea* metabolic model

Nitrogen starvation causes a re-routing of carbon fluxes in microalgae leading to a gradual decrease in the carbon fixation rate. The original metabolic model proposed by Baroukh et al. (2014), which was developed for nutrient replete conditions, represents a slowdown of photosynthesis (due to the decrease of functional biomass resulting from C accumulation). But this was not enough: excretion clearly improves model fitting and its predictive capability. It allows a good representation of the carbon fluxes, especially in case of substrate limitation or starvation, by limiting the accumulation of carbon and adapting it to the structural biomass. This confirms the key role of excretion in microbial metabolism and its modeling, although it is generally not considered [except in the recent works for *Prochlorococcus* of Ofaim et al. (2021)].

Different excreted molecules (namely EPS, acetate, and glycerol) have been tested *in silico* and EPS seem to be the most likely. Other excretion schemes associated to other metabolites cannot be excluded, and considering the similar performances for the four studied cases (Figures 3, 7), it is likely that they would also lead to plausible predictions. Additional experiments are definitively required to better characterize the nature of the excreted molecules and the associated fluxes, so as to definitely select the most appropriate model (potentially implying several excreted molecules). More generally, some metabolic fluxes are well characterized (e.g., the photosynthetic reactions R1-14), while others suffer from larger uncertainties (see Supplementary File S2). Measurements of targeted intracellular fluxes would be highly valuable to reduce the uncertainties on model parameters and, *in fine*, on the intracellular fluxes and biomass dynamics.

A key issue that our model addresses is the quantification of carbon excretion. This is necessary to expand our understanding of microbial interactions within the phycosphere (Seymour et al., 2017) or more broadly the microbial loop in the ocean (Azam et al., 1994; Pomeroy et al., 2007). A full grasp of excretion is also required to better predict primary production, as recently pointed out by Wu et al. (2021) using a biogeochemical model in which photosynthesis and biosynthesis are decoupled. Our model predicts that the percentage of excretion decreases with growth rate, which is in line with field observations, that is excretion is higher in oligotrophic ocean than in productive zones (such as upwelling regions) (Moran et al., 2022). Quantitatively, this survey shows that excretion represents around 25% of the net primary production (ranging from 3 to 50%). More specifically, for *T. lutea*, Claquin et al. (2008) report that 15.9% of the photosynthetic carbon production was excreted in non-limiting condition. This value is based only on transparent exopolymeric particle (TEP) measurements and thus probably underestimates the actual excreted photosynthetic carbon. Considering all this, the ExCarb model gives the soundest predictions in term of excretion (e.g., 25% in non-limiting condition), but our models tend to globally overestimate this flux.

### 3.2. Other mechanisms at play

While excretion clearly improves model predictions and is likely to play a central role, other mechanisms – not considered herein – are known to mitigate nutrient stress. The most important one is probably the dissipation of light energy. The synthesis of photoprotective pigments, such as carotenoids, can help to dissipate the excessive light energy received by the cell during nitrogen starvation (Stehfest et al., 2005; Solovchenko et al., 2013). Indeed, some of these photoprotective pigments can perform non-photochemical quenching (NPQ) *via* the xanthophyll cycle, which harmlessly dissipate excess excitation energy as heat through molecular vibrations (Niyogi et al., 1997). Other mechanisms, including dissipation of electrons, NADPH, ATP and carbon *via* several pathways (Melher-like reactions, alternative electron flow, photorespiration, futile cycles...), might take place to dissipate the excess of incoming energy.

These phenomena are of course non-exclusive and are likely to take place simultaneously to more efficiently address different time scales of light or nutrient variations. As none of these dissipating mechanisms were directly or indirectly measured during the experiment, it is difficult to know whether they take place and at which extent. Additional experiments with supplementary measurements, such as the profile of the carbon pools in the cell and of the excreted carbon, are definitely required. They will allow to more accurately close the carbon and energy balances, and eventually to unravel which of these mechanisms takes place, and even quantify their respective effects.

### 3.3. New insights on the Droop function

Our results now provide new insights about the Droop function, which is largely used to describe phytoplankton growth (Droop, 1968, 1983). The Droop function is an empirical function representing the effect of the limiting nutrient internal quota *q* (N/C in our case) on phytoplankton specific growth rate μ(*q*), see Equation (1).

**Figure 8:**
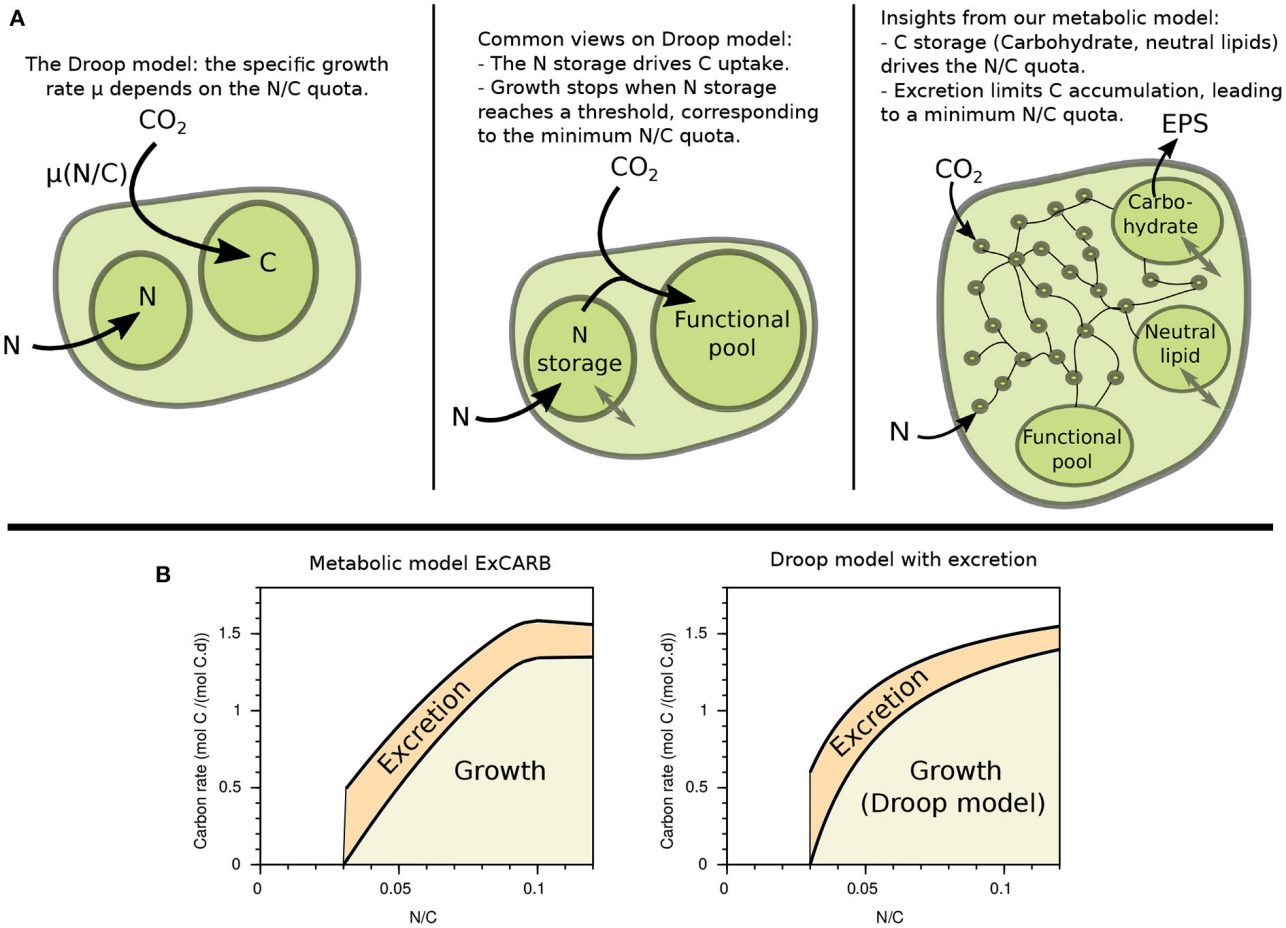
Revisiting Droop model. **(A)** Illustrations of the empirical relationship (left) and common views (middle) on Droop model, in comparison with the insight from our metabolic model (right). **(B)** Growth and excretion rates as a function of the N/C quota given by the metabolic model ExCARB (left) and by the Droop model (2) with the additional equation (3) (right).

Droop model is generally justified by the necessity for the cell to have stored enough limiting element to grow (see Figure 8). Note that there is often a confusion in the literature, where “internal quota” is often mixed up with “storage pool”, so that it is sometimes written that the growth rate depends on the internal nitrogen storage. Such vision [implemented in Lemesle and Mailleret (2008)] would provide a clearer mechanistic explanation, but this is not the Droop model. The quota model principle is meaningful at a macroscopic level, but its interpretation at the level of metabolism is not clear. In our metabolic model, we assume that carbon and nitrogen uptakes are respectively triggered by light intensity and nitrate concentration. This uncoupling induces the observed variable N/C ratio in our simulations, which was further validated with experimental data. This makes a strong difference with bacteria where in general carbon and nitrogen uptakes are tightly coupled (Doucette et al., 2011), explaining why these microorganisms are overall less plastic than microalgae. Paradoxically, the variable N/C in our simulations is not due to the storage of the limiting nutrient (N in our case), but rather to variable carbon storage accumulation. This interpretation, already sometimes used within macroscopic models (Mairet et al., 2011; Wu et al., 2021; Di Caprio, 2022), inverses the classical point of view on the Droop model. It means that the represented variable growth rate in the Droop model would be due to the storage of carbon in the cell rather than to the intracellular storage of the limiting element (see Figure 8). This feature explains the ability of the metabolic models including excretion to link the specific growth rate to the internal quota. Our modeling approach highlights that the minimum quota for growth actually corresponds to a maximum accumulation of carbon storage. This limit appears in our simulations because of excretion, but it can also be due to physical constraints inside the cell.

There is a growing body of literature showing that excretion increases with nutrient limitation (Moran et al., 2022), as we also see in our simulations. The Droop model, which represents only biomass, expresses the specific growth rate as a function of the internal quota *q*:


(2) $$\begin{array}{r} \mu \left(q\right)=\mu_{m}\left(1-\frac{Q_{0}}{q}\right) \end{array}$$

It can be complemented with an excretion rate η (expressed in term of mass of carbon excreted per unit of carbon biomass and time). Given the linear trend observed between the excretion and the growth rate in Figure 7, we propose a simplified model which completes the Droop model by representing the flux of excreted carbon. In this add-on model, η depends, as the growth rate, on the inverse of the nutrient quota:


(3) $$\eta (q)=\bar{\eta }\frac{Q_{0}}{q},$$

where $\bar{\eta }$ is the maximum excretion rate, obtained for *q* = *Q*_0_. This choice of kinetics becomes more straightforward when the quota is flipped: the excretion rate η actually increases linearly with the C/N ratio, i.e., with carbon accumulation. A comparison of this model with the output of the ExCARB model is given in Figure 8.

The Net Primary Production can now be estimated by summing biomass growth and excretion:


(4) $$\begin{array}{c} \mu (q)+\eta (q)=\bar{\mu }[1-(1-\frac{\bar{\eta }}{\bar{\mu }})\frac{Q_{0}}{q}]. \end{array}$$

Additional experiments at different levels of nutrient limitation are now necessary to further validate this coarse grain model. It will eventually contribute to propose a more accurate picture of the carbon fluxes at the global scale.

In conclusion the set of considered metabolic models gives a mechanistic view on the Droop model during nitrogen stress, and explains why this simple model is very efficient, as demonstrated by Mairet et al. (2011). Surprisingly, Droop model has been shown to efficiently represent limitation with very different types of nutrients, from a co-factor (vitamin B12) to a key constituent of proteins (nitrogen) or energy carriers (phosphorus). Whether our mechanistic explanation (with nitrogen stress) can be extrapolated to other limiting nutrients is not straightforward, given the different roles they have. However, in any case, the limiting nutrient slows down the production of functional biomass, so that the incoming carbon flux has to be redirected toward carbon storage (Fernandes et al., 2013). The same alternative explanations of an actual carbon storage might therefore also reveal these different behaviors, and deserves to be further consolidated by dedicated studies.

## 4. Materials and methods

### 4.1. Experimental data

The experimental data of Lacour et al. (2012a) and Lacour et al. (2012b) were used to support respectively the model calibration and validation. In brief, in both studies, *T. lutea* (clone T-iso, CCAP 927/14) was grown in duplicate chemostat in 5 L cylindrical vessels at constant temperature (22°C) and pH (8.2, maintained by automatic injection of CO_2_). For model calibration (Lacour et al., 2012a), light intensity I(t) was monitored to mimic a 12 h light — 12 h night cycle, with approximately 1,500 μmol.m^−2^.s^−1^ measured at noon at the center of the reactor. The experiment was carried out for 8 days, and a nitrogen starvation was performed from day 1 to day 5.5 (Supplementary Figure S1). Nitrogen starvation was achieved by removing nitrates in the incoming media, and waiting for the complete exhaustion of nitrates in the chemostat. At day 5.5, nitrate was reintroduced, under the form of a pulse (2.7 mgN.L^−1^) and simultaneously added in the incoming media. Since growth is hindered during nitrogen starvation, the dilution rate was decreased accordingly in order to avoid washout. Nitrate, particulate carbon and nitrogen, chlorophyll, total carbohydrates and neutral lipid concentrations were measured throughout the experiment (Lacour et al., 2012a). The latter two were expressed as quota, i.e., divided by particulate carbon.

For model validation (Lacour et al., 2012b), nitrogen-limited chemostat cultures under constant light (at 430 μmol.m^−2^.s^−1^) were carried out. Different levels of nitrogen stress were obtained through a succession of dilution rate changes. Steady-states values of nitrogen quota and carbon storage quota were used for model validation.

### 4.2. Model equation

The metabolic model of *T. lutea* has been reduced to seven macro-reactions given in Table 1 [see Baroukh et al. (2014)], with additionally three different excreted molecules. For carbohydrates, the EPS excretion is made directly from the CARB pool. For acetate, excretion is only a few reaction steps from PEP, since acetate is synthesized from Acetyl-CoA:


$$\begin{array}{l} \text{ ADP}+\text{H}+\text{PEP}\to \text{PYR}+\text{ATP}\\ \text{PYR}+\text{NAD}+\text{CoA}\to \text{AcCoA}+\text{CO2}+\text{NADH}\\ \text{ AcCoA}+\text{Pi}+\text{ADP}↔\text{ACE}+\text{ATP}+\text{CoA} \end{array}$$

Similarly, glycerol is only a few reaction steps from GAP, since glycerol is synthesized from DHAP:


$$\begin{array}{l} \text{ GAP}↔\text{DHAP}\\ \text{ DHAP}+\text{H2O}↔\text{DHA}+\text{Pi}\\ \text{DHA}+\text{H}+\text{NADPH}↔\text{GLYC}+\text{NADP} \end{array}$$

These reactions are gathered into a macro-reaction given in Table 1.

The dynamical model, obtained from mass balance, is described by an ODE system representing the dynamics over time of substrate S (nitrate), accumulating metabolites A (GAP, PEP, G6P, PA, CARB), functional biomass B and excreted molecule P (in mol/L):


(5) $$\begin{array}{c} \frac{d}{dt}\left(\begin{array}{c} S\\ A\\ B\\ P \end{array}\right)=K^{\prime }\alpha B+D\left(\begin{array}{c} S_{in}\\ 0\\ 0\\ 0 \end{array}\right)-D\left(\begin{array}{c} S\\ A\\ B\\ P \end{array}\right) \end{array}$$

with *S*_*in*_ the incoming substrate concentration and D the dilution rate. The composition of functional biomass is determined from experimental data (Lacour et al., 2012a). The stoichiometric matrix *K*′ and the kinetic vector α result from Table 1. In addition, total biomass, in terms of particulate carbon *X*_*C*_ and nitrogen *X*_*N*_ (in mol/L), is computed using a mass balance:


$$X_{C}(t)=\underset{A}{\sum^{\text{​}}}C_{A}A(t)+C_{B}B(t)$$

$$X_{N}(t)=\underset{A}{\sum^{\text{​}}}N_{A}A(t)+N_{B}B(t)$$

where *C*_*A*_ and *C*_*B*_ correspond to the number of carbon atoms per molecule of A and B and *N*_*A*_ and *N*_*B*_ correspond to the number of nitrogen atoms per molecule of A and B. Metabolic fluxes of the cell can be computed using the elementary flux mode matrices that were obtained during the reduction of the 162 reactions of metabolic network to macroscopic reactions. That is, the flux of reaction *j* is given by:


$$v_{j}(t)=\underset{i}{\sum^{\text{​}}}a_{ij}\alpha_{i}(t),$$

where *a*_*ij*_ is the contribution of reaction *j* in the macro-reaction *i*, given by the elementary flux mode matrices. The consumption and production of ATP and NADPH are computed from these fluxes to check a posteriori their balance, e.g., for ATP:


$${\text{Cons}}_{\text{ATP}}(t)=-\underset{j}{\sum^{\text{​}}}\min(0,K_{i_{\text{ATP}},j}v_{j}(t)),$$

$${\text{Prod}}_{\text{ATP}}(t)=\underset{j}{\sum^{\text{​}}}\max(0,K_{i_{\text{ATP}},j}v_{j}(t)),$$

where *K* is the stoichiometric matrix of the core network. For further details on this model construction, the reader is referred to Baroukh et al. (2014).

### 4.3. Calibration strategy

*T. lutea* cultures under nitrogen starvation and day/night cycles (Lacour et al., 2012a) were used for model calibration. For each model, parameters were estimated by minimizing the sum of squared error (SSE) between simulation and experimental measurements. The Nelder-Mead algorithm implemented in the Scilab software (fminsearch) was used. In line with the experimental protocol, the simulations were started 5 days before the beginning of the measurements to reach a periodic regime (i.e., simulations start at *t*_0_ = −5d). The simulation outputs as shown on Figure 3 are thus not depend on the initial conditions. A constraint was added on *k*_MR1_ to account for the quantum limit of photosynthesis. Using an absorption coefficient of 16 m^2^/g Chl (Bannister, 1974) and a photosynthetic requirement of 10 photons per fixed carbon (Baroukh et al., 2014), we get an upper bound of 4.5 10^−3^ μE^−1^.m^2^.s.h^−1^.mol B^−1^. To reduce the risk of ending up in a local minima, several optimizations were performed with random initial parameters. The parameters were re-estimated for each model since the extension is likely to modify the distribution of fluxes between the different metabolic branches. For example, if excretion is performed at the level of carbohydrates, fluxes in upper glycolysis should be higher so as to compensate carbon loss. The w/oEx model was calibrated with nitrogen replete condition (the first day of measurements), as already done in Baroukh et al. (2014). Alternatively, a set of parameter minimizing the error on the whole experiment (including nitrogen starvation) was also determined, and the resulting model calibration was called w/oEx*. For the four extended models (ExCARB, ExG6P, ExPEP, ExGAP), the same parameters estimation procedure was carried out using the whole experiment. Results of parameter identification are presented in Table 2. Confidence intervals on model parameters have been estimated following Casagli et al. (2021). Briefly, the Fisher Information Matrix (FIM) is computed from the sensitivity functions of model outputs *y* with respect to parameters θ and the covariance matrix of measured standard deviation *W*:


$$\begin{array}{l} FIM=\underset{i}{\sum }\left(\frac{\partial y\left(t_{i}\right)}{\partial \theta }\right)W\left(t_{i}\right){\left(\frac{\partial y\left(t_{i}\right)}{\partial \theta }\right)}^{T} \end{array}$$

The standard deviations on model parameters are then computed from the diagonal terms of the FIM's inverse:


$$\begin{array}{l} \sigma_{\theta_{j}}^{2}={\left(FIM^{-1}\right)}_{j,j}. \end{array}$$

From these values, we can estimate the error propagation on all the model outputs (i.e., the biochemical concentrations and the intracellular fluxes), as follows:


$$\begin{array}{l} \sigma_{y_{i}}^{2}\left(t\right)=\underset{j}{\sum }{\left(\frac{\partial y_{i}\left(t\right)}{\partial \theta_{j}}\right)}^{2}\sigma_{\theta_{j}}^{2}. \end{array}$$

The 95% confidence intervals on model outputs are finally given by *y*_*i*_(*t*)±1.96σ_*y*_*i*__(*t*).

To compare models with different parameter numbers, the corrected Akaike Information Criterion (AICc) was computed as follows (Burnham and Anderson, 2002):


$$AIC_{c}=n\log\left(\frac{SSE}{n}\right)+2\left(p+1\right)+\frac{2\left(p+1\right)\left(p+2\right)}{n-p-2}$$

where *n* is the number of measurements, and *p* the number of estimated parameters. The model with the lowest AICc should be preferred.

### 4.4. Model validation

Steady-state chemostat cultures under nitrogen limitation at various dilution rates (Lacour et al., 2012b) were used to validate the models. Simulations with dilution rates ranging from 0.02 d^−1^ to 1.4 d^−1^ were carried out. For all the conditions, nitrogen and carbon storage quotas were computed when the trajectories reach their equilibria. Several randomly chosen initial conditions were considered to check that the equilibria do not depend on them. The squared-error between simulation and experimental measurements were computed to assess model prediction capability. The Droop model (Equation 1) was fitted to the experimental data using the least-squares routine *leastsq* in Scilab.

## Data availability statement

The original contributions presented in the study are included in the article/Supplementary material, further inquiries can be directed to the corresponding author.

## Author contributions

OB and CB: conceptualization. CB and FM: methodology, formal analysis, investigation, writing—original draft, and visualization. OB: resources, project administration, and funding acquisition. CB, FM, and OB: writing—review and editing. All authors contributed to the article and approved the submitted version.

## Funding

This work was supported by the ANR project PhotoBiofilm explorer (ANR-20-CE43-0008) and by the FMJH Program PGMO (and from the support to this program from EDF-THALES-ORANGE). Work of CB is supported by the French Laboratory of Excellence project TULIP (grant number ANR-10-LABX-41; ANR-11-IDEX- 0002-02).

## Conflict of interest

The authors declare that the research was conducted in the absence of any commercial or financial relationships that could be construed as a potential conflict of interest.

## Publisher's note

All claims expressed in this article are solely those of the authors and do not necessarily represent those of their affiliated organizations, or those of the publisher, the editors and the reviewers. Any product that may be evaluated in this article, or claim that may be made by its manufacturer, is not guaranteed or endorsed by the publisher.

## Supplementary material

Supplementary Figure S1 Experimental conditions for the calibration experiment, from Lacour et al. (2012a).

Supplementary Figure S2 Comparison of model uncertainties between the w/oEx* and ExCARB models. The thin lines represent the 95% confidence intervals.

Supplementary File S1 Scilab code for model simulation.

Supplementary File S2 Reaction fluxes (and their standard deviations) for all the models under non-limiting condition (*t* = 0.75 d) and starvation (*t* = 3.75 d).

## Abbreviations

PG: 6-Phosphogluconate
AcCoA: Acetyl-CoA
ACE: Acetate
AKG: alpha-ketoglutarate
B: Functional Biomass
CARB: Carbohydrate
E4P: Erythrose 4-phosphate
EPS: Exopolysaccharides
F6P: Fructose 6-phosphate
FUM: Fumarate
G3P: Glycerate 3-phosphate
G6P: Glucose 6-phosphate
GAP: Glyceraldehyde 3-phosphate
GLYC: Glycerol
ICIT: Isocitrates
MAL: Malate
OA: Oxaloacetate
PA: Phosphatic Acid
PEP: Phosphoenolpyruvate
Pi: Orthophosphate
PYR: Pyruvate
R5P: Ribose 5-phosphate
Ru5P: Ribulose 5-phosphate
RuBP, Ribulose 1: 5-bisphosphate
S7P: Sedoheptulose 7-phosphate
SUC: Succinate
X5P: Xylulose 5-phosphate
Xc: Total biomass.
