# Supplementary figures and images for: The paradoxes hidden behind the Droop model highlighted by a metabolic approach
Source: Front Plant Sci. 2022 Aug 22;13:941230. doi: 10.3389/fpls.2022.941230 (PMC9442053; doi:10.3389/fpls.2022.941230)

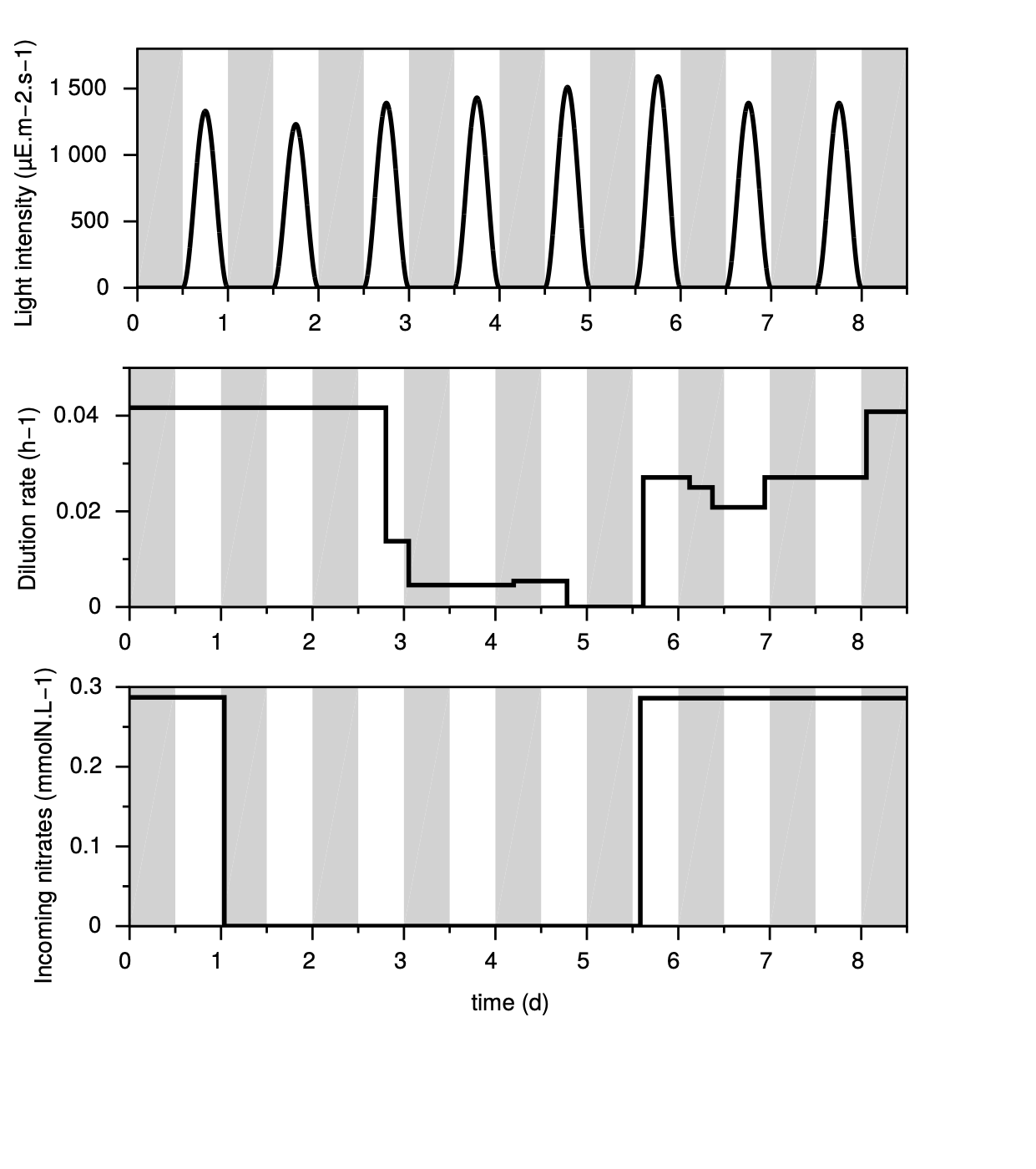

Supplement: Supplementary Figure S1 — Experimental conditions for the calibration experiment, from Lacour et al. (2012a). [file Image_1.TIFF]
